# LncRNA MSTRG.22719.16 mediates the reduction of enoxaparin sodium high-viscosity bone cement-induced thrombosis by targeting the ocu-miR-326-5p/CD40 axis

**DOI:** 10.1186/s13018-023-04109-5

**Published:** 2023-09-22

**Authors:** Linchao Sang, Luobin Ding, Kangning Hao, Ce Zhang, Xiaoyu Shen, Hui Sun, Dehao Fu, Xiangbei Qi

**Affiliations:** 1 Department of Orthopaedic Surgery, The Third Hospital of Hebei Medical University, Shijiazhuang, China; 2 Department of Orthopaedic Surgery, Faculty of Medicine, Oita University, Oita, Japan; 3 Department of Orthopaedics, Shanghai General Hospital, Shanghai Jiao Tong University School of Medicine, Shanghai, 200080 People’s Republic of China

**Keywords:** Thrombosis, Enoxaparin sodium, Endothelial cells, High-throughput RNA sequencing, CeRNA

## Abstract

**Objective:**

Polymethylmethacrylate (PMMA) bone cement promotes the development of local thrombi. Our study found that a novel material, ES-PMMA bone cement, can reduce local thrombosis. We used a simple and reproducible animal model to confirm the reduction in local thrombosis and explored the associated molecular mechanism.

**Methods:**

New Zealand rabbits, which were used to model thrombosis using extracorporeal carotid artery shunts, were divided into the following two groups, with 3 rabbits in each group: the PMMA bone cement group and the ES-PMMA bone cement group. Four hours after modelling, experimental samples, including thrombotic and vascular tissues, were collected. Thrombotic samples from the PMMA group and ES-PMMA group were subjected to lncRNA sequencing, and a lncRNA microarray was used to screen the differentially expressed lncRNAs. The expression of thrombomodulin in endothelial cells was quantified in vascular tissue samples. Differences in the lncRNA expression profiles between the thrombotic samples of the PMMA group and ES-PMMA group were assessed by base-to-base alignment in the intergenic regions of genomes. The lncRNA-miRNA-mRNA competitive endogenous RNA (ceRNA) network was established in light of ceRNA theory. Thrombosis was observed in the PMMA group and ES-PMMA group.

**Results:**

The thrombotic weight was 0.00706 ± 0.00136 g/cm in the PMMA group and 0.00551 ± 0.00115 g/cm in the ES-PMMA group. Quantitative real-time polymerase chain reaction (RT–q-CR) and Western blotting revealed that the expression of CD40, which can regulate thrombosis in vascular endothelial cells, was significantly lower in the ES-PMMA group than in the PMMA group. High-throughput sequencing was used to identify 111 lncRNAs with lower expression in the ES-PMMA group than in the PMMA group. Through bioinformatics investigation, lncRNA MSTRG22719.16/ocu-miR-326-5p/CD40 binding sites were selected. Fluorescent in situ RNA hybridization (FISH) was performed to verify the lower expression of lncRNA MSTRG.22719.16 in vascular tissues from the ES-PMMA group. A dual-luciferase reporter gene assay was applied to verify that ocu-miR-326-5p binds the CD40 3ʹ-UTR and targets lncRNA MSTRG.22719.16.

**Conclusion:**

Compared with PMMA bone cement, ES-PMMA bone cement can reduce thrombosis through the lncRNA MSTRG.22719.16/ocu-miR-326-5p/CD40 axis.

**Supplementary Information:**

The online version contains supplementary material available at 10.1186/s13018-023-04109-5.

## Introduction

Clinicians have used bone cement for a long time, and with the continuous development of joint replacement strategies, complications and adverse events have gradually emerged. Currently, bone cement is divided into two types: high-viscosity bone cement used in hip and knee arthroplasty and low-viscosity bone cement. The incidence of bone cement implantation syndrome (BCIS), an important and severe complication, is as high as 28% [1]. However, the exact mechanism of BCIS has not yet been fully determined. At present, researchers believe that BCIS is mainly caused by pulmonary embolism, allergic reactions, histamine release and complement activation combined with ventilation/perfusion mismatch and an increase in pulmonary vascular resistance, which eventually leads to acute cardiogenic shock, right ventricular failure and hypoxia [2]. The mortality rate is very high, although pulmonary embolism is rare after joint replacement. According to previous reports, the exact incidence of pulmonary embolism after joint replacement may be as high as 0.2–0.4% [3].

In the current study, we examined the toxicity of bone cement particles in the blood, as thrombosis results from the coagulation system due to thermal effects and complement activation [4]. Thrombus shedding can lead to pulmonary embolism or extremity venous thrombosis. Our previous studies showed that the physical and chemical properties of bone cement can directly trigger thrombosis [5].

Among orthopaedic patients, enoxaparin sodium is widely used to prevent and treat thrombosis-related diseases [6]. Enoxaparin sodium high-viscosity bone cement is a biomaterial made by mixing bone cement powder with enoxaparin sodium powder in a certain proportion and then mixing the product with liquid [7].

The results of our previous studies also showed that high-viscosity bone cement can induce local thrombosis in animal models and that compared with ordinary bone cement, enoxaparin sodium high-viscosity bone cement, a novel material, can reduce local thrombosis [5]. The mechanisms leading to thrombosis are as follows: slowing of blood flow caused by prolonged bed rest [8]. A hypercoagulable state caused by inflammation [9] and abnormal endothelial cell function due to endothelial injury [10]. The proteins associated with thrombosis in endothelial cells include endothelins [11, 12], vascular cell adhesion molecule [13, 14], CD62p [15, 16], CD31 [17, 18], thrombomodulin [19, 20] and CD40 [21–23]. Long noncoding RNA (lncRNA) belongs to a class of noncoding RNAs longer than 200 nucleotides. LncRNAs play a crucial role in many processes, including dose compensation, epigenetic regulation, and cell cycle control and differentiation. LncRNA expression plays a critical role in the initiation and progression of thrombosis [24–26]. These molecules are crucial regulators of endothelial cell function. Therefore, the underlying mechanisms by which enoxaparin sodium high-viscosity bone cement reduces thrombotic formation need to be unravelled.

To quantitatively assess thrombosis in animal models [27–29], we successfully established animal models that can quantitatively measure the thrombosis induced by high-viscosity bone cement and collected thrombus samples for RNA sequencing to examine the differential expression of lncRNAs. By comparing the lncRNA expression profiles from the two thrombosis groups, we identified differentially expressed lncRNAs. On this basis, the lncRNA MSTRG.22719.16/ocu-miR-326-5p/CD40 axis was identified. The proposed mechanism of ceRNA-based regulation has been experimentally validated in processes such as the regulation of thrombosis. As described in detail below, we elucidate the molecular mechanism by which this material reduced local thrombosis.

## Methods

### Animal model, grouping and experimental reagents

**Fig. 1:**
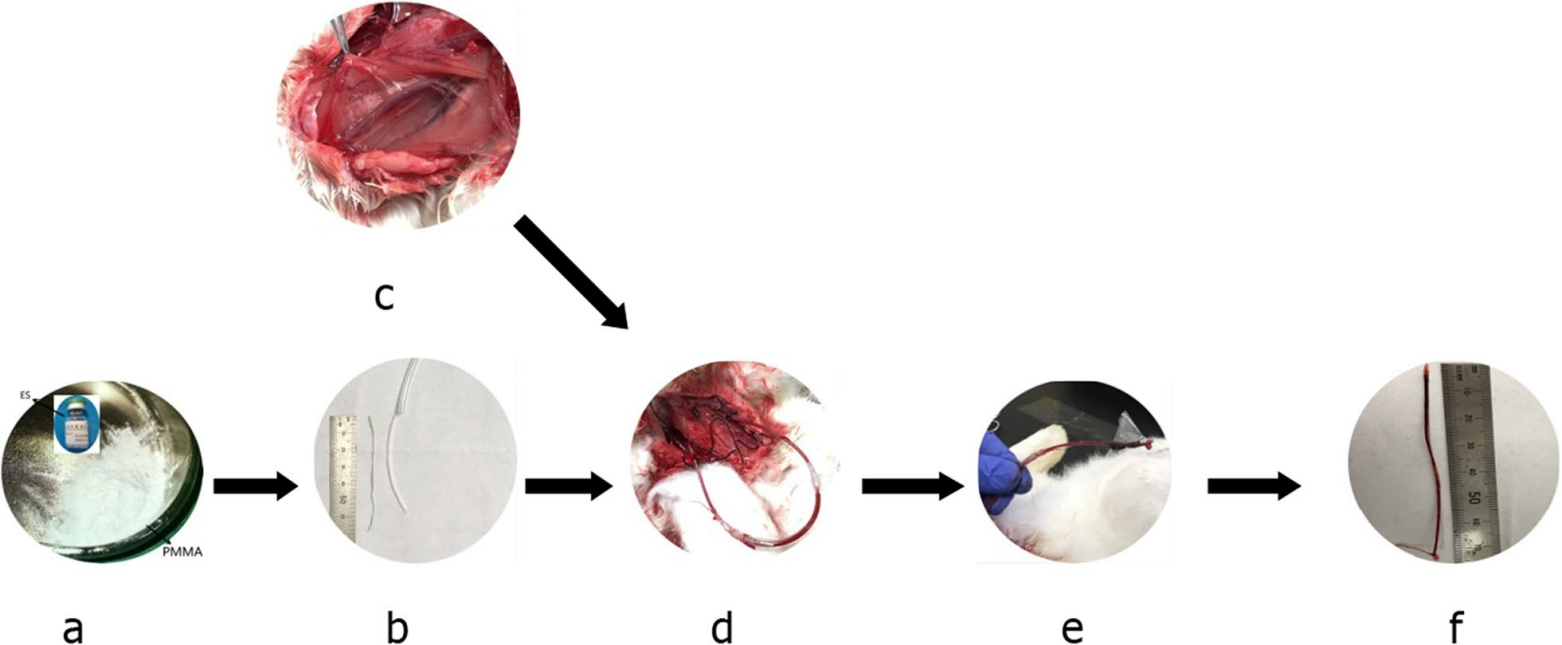
**a** High-viscosity bone cement powder and enoxaparin sodium (ES) samples. **b** The extracorporeal shunt and prepared silk thread with bone cement. **c** The exposed arteriovenous vessels of New Zealand rabbits. **d** Establishment of the animal model. **e** Blood vessels contacting the thread covered with bone cement. **f** Intravascular bone cement-induced thrombus samples

**Fig. 2:**
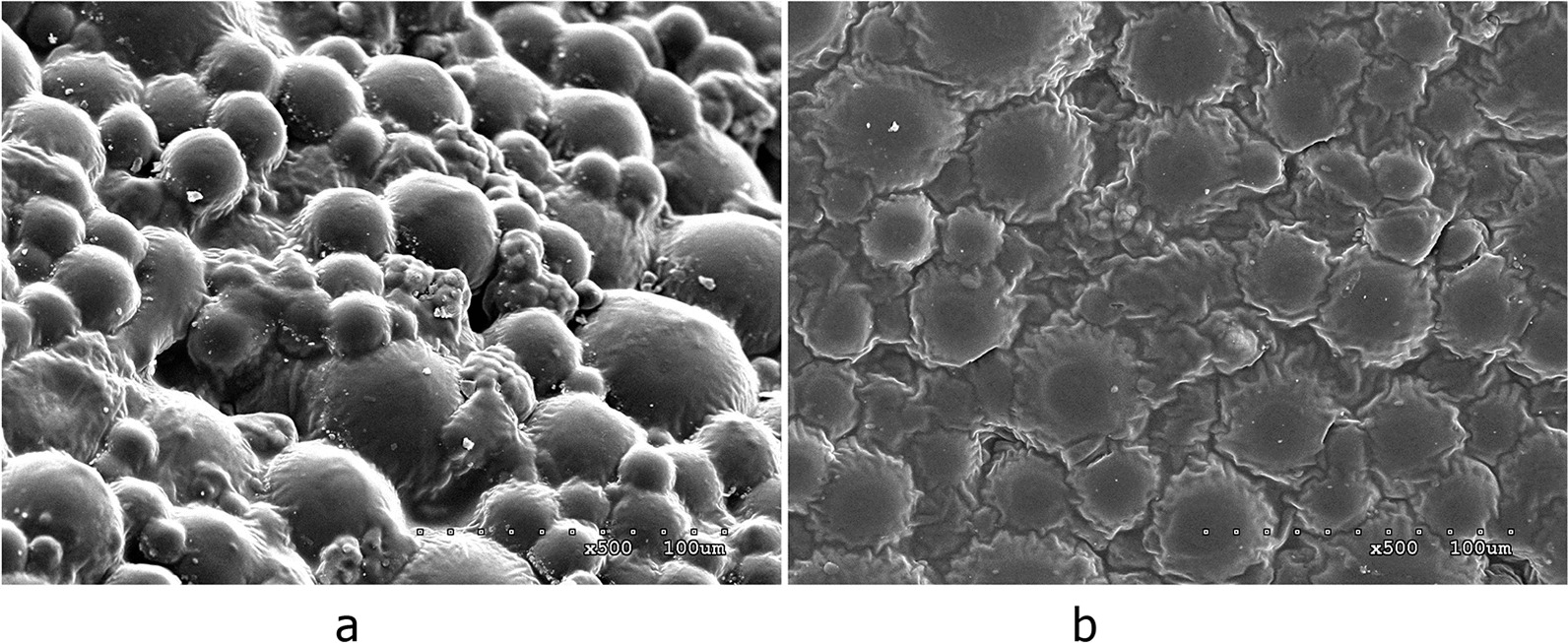
**a** PMMA. There were many gaps between the bone cement particles. **b** ES-PMMA. The surface of the bone cement particles was covered with a syrup-like substance, which was considered the attached enoxaparin

Six-month-old male New Zealand rabbits (Wangdu Tonghui Animal Breeding Co., Ltd., animal certificate no. 210426) (protocol approved by the Medical Ethics Committee of the Third Medical College of Hebei Medical University, ethics acceptance no. z2021-005-2 and the Third Hospital of Shijiazhuang ethics acceptance no. 2020–039) weighing 2.5 ± 0.5 kg were anaesthetized. An appropriate depth of anaesthesia was maintained with 20% urethane. An extracorporeal carotid artery shunt was placed as described in the literature [29]. The rabbits were divided into two groups: the novel enoxaparin sodium high-viscosity polymethylmethacrylate (ES-PMMA) bone cement group (M group) (*n *= 3) and the ordinary high-viscosity polymethylmethacrylate (PMMA) bone cement group (Con group) (*n *= 3). For generation of ES-PMMA, 8000 AXa IU enoxaparin sodium powder (Chengdu Baiyu, China) was premixed with 40 g PMMA bone cement (Heraeus, Germany) [7], followed by addition of liquid. Then, the mixture was placed under a high-power scanning electron microscope (SEM, Hebei Medical University Electron Microscopy Center, Hitachi, S-3500N), and the characteristics were compared. We ensured that the bone cement completely and evenly covered a surgical silk thread. Then, the silk thread was inserted into the shunt and extended approximately 1 cm into the blood vessel at the same time. The procedures used for sample collection and modelling are shown in Fig. 1, and the characteristics of ES-PMMA and PMMA are shown in Fig. 2.

### Sample collection

We collected and analysed samples 4 h after modelling. Thrombus samples were obtained, and the degree of thrombus attached to the thread was determined. High-throughput sequencing was used to analyse lncRNA expression in two groups of thrombotic samples. Vascular tissue samples that contacted the thread were stored in a − 80 °C freezer. The expression of thrombus-related regulatory proteins in endothelial cells was determined by Western blot and quantitative real-time polymerase chain reaction (RT-qPCR).

### RT-qPCR analysis of the mRNA expression of thrombus-associated proteins in vascular tissue

Total RNA was extracted from tissues with TRIzol reagent (Invitrogen, USA) according to the manufacturer's instructions. The mRNA was reverse transcribed with a PrimeScript RT Reagent Kit (TaKaRa, Japan). A SYBR Premix Ex Taq Kit (Servicebio, Wuhan) was used for RT-qPCR on an iQ5 Real-Time PCR instrument (Applied Biosystems USA). The reaction conditions were as follows: 94 °C for 4 min, 94 °C for 30 s, 60 °C for 30 s and 72 °C for 30 s for a total of 40 cycles. Each sample was analysed three times. The primer sequences for the PCR were as follows: CD31: F, TCCTACGATGCCAGGTCTGA, and R, CATTTCGGCATGGGAATGGC; CD40: F, GGCGGGAACTAACAAGACAG, and R, GCGGTAGCCCTTATCTATTGG; CD62p: F, AGTGTGTAGCTGTCCAGTGC, and R, AGTCACCAAAGGGATGCGAG; CD106: F, GCCCTTTGGAGGTTGGAGAA, and R, GAACTGGTAGACCCTCGCTG; endothelin: F, TGACTCCCAGAGAGGACGTG, and R, CTCCTGGACGGCTACAATCC; thrombomodulin: F, TTCCTCTGCGAGTTCCCCTT, and R, CGTAACAGGTCAGCTCCAAG; and GAPDH: F, TGGAATCCACTGGCGTCTTC, and R, TCATGAGCCCCTCCACAATG. The 2^−ΔΔCt^ method was applied for relative quantitative analysis, and a histogram was drawn. Gene expression was normalized to GAPDH expression.

### Western blot analysis

Blood vessel samples in the different groups were washed with ice-cold PBS and then lysed with RIPA buffer (Beyotime, China) containing protease inhibitor. The protein samples were separated on SDS-PAGE gels of different percentages and then transferred to a polyvinylidene fluoride (PVDF) membrane (Millipore, USA). The membrane was incubated with primary antibodies against CD31 (Abcam, UK), CD40 (Abcam, UK), CD62P (Abcam, UK), CD106 (Abcam, UK), endothelin (Abcam, UK) and thrombomodulin (Abcam, UK) at 4 °C with shaking overnight and then with a corresponding secondary antibody for 1.5 h. Western LightningTM Chemiluminescence Reagent was used to develop the blot for 30 s, and then, the membrane was immediately placed in an exposure box and exposed for 1 min in a darkroom. The membrane was imaged and analysed with a LabWorksTM gel imaging and analysis system (UVP, USA). GAPDH was used as the internal control.

### LncRNA high-throughput sequencing analysis

A total RNA isolation kit (TR205-200, Tianmo, CN) was used to extract the total RNA from thrombotic samples according to the instructions provided by the manufacturer. In this project, all experimental procedures followed the standard protocols provided in the product manuals. The Agilent Bioanalyzer 2100 system (Agilent Technologies, CA, USA) was used to measure RNA integrity. Our measurements of RNA concentration and purity were performed using a Qubit® 3.0 Fluorometer (Life Technologies, CA, USA) and Nanodrop One spectrophotometer (Thermo Fisher Scientific, Inc., USA). Every group was analysed with three independent samples. In Fig. 6, we show the results. We used version OryCun2.0.102 of the rabbit genome as our reference, and the data were analysed by aligning sequencing reads to genome reference sequences using the software HISAT2. In this section, we will discuss how the algorithm described above can be adapted to align spliced sequences. Each gene's number of read genes, including new genes, was counted. The results are represented in Fig. 7. StringTie software was used to count the fragments within each gene, and the trimmed mean of M values (TMM) algorithm was used for normalization. As a measure of total gene expression, we used the fragments per kilobase of exon per million reads mapped (FPKM). A sample-specific transcriptome composed of transcripts assembled by StringTie was constructed using gffcompare (https://github.com/gpertea/gffcompare). For unaligned transcript sequences, it is necessary to predict new genes and new long noncoding RNAs and to predict whether they might be new genes or lncRNAs. The results are shown in Fig. 8. The ceRNA network of CD40 was predicted by merging all ceRNA-ceRNA interactions of each gene expression profile. The resulting value is represented as shown in Fig. 9 and Table 1.

**Table 1:** Numeric scale of CD40 gene ceRNA-regulated pathways

mRNA	miRNA	lncRNA	Sum max energy
CD40	ocu-miR-370-3p	MSTRG.4956.8	− 60.68
CD40	ocu-miR-370-3p	MSTRG.52539.1	− 61.17
CD40	ocu-miR-370-3p	MSTRG.4956.9	− 60.68
CD40	ocu-miR-370-3p	MSTRG.20869.3	− 61.17
CD40	ocu-miR-370-3p	MSTRG.86591.5	− 60.96
CD40	ocu-miR-146b-3p	MSTRG.79535.1	− 63.18
CD40	ocu-miR-146b-3p	MSTRG.73665.1	− 68.1
CD40	ocu-miR-146b-3p	MSTRG.71816.1	− 66.89
CD40	ocu-miR-146b-3p	MSTRG.86903.8	− 64.3
CD40	ocu-miR-12093-3p	MSTRG.85304.2	− 61.78
CD40	ocu-miR-12093-3p	MSTRG.85304.1	− 61.78
CD40	ocu-miR-326-5p	MSTRG.8098.12	− 70.85
CD40	ocu-miR-326-5p	MSTRG.86533.1	− 66.5
CD40	ocu-miR-326-5p	MSTRG.33446.2	− 63.87
CD40	ocu-miR-326-5p	MSTRG.13150.1	− 64.86
CD40	ocu-miR-326-5p	MSTRG.85249.38	− 63.48
CD40	ocu-miR-326-5p	MSTRG.8098.11	− 70.85
CD40	ocu-miR-326-5p	MSTRG.71739.1	− 69.33
CD40	ocu-miR-326-5p	MSTRG.22719.16	− 66.54
CD40	ocu-miR-326-5p	MSTRG.22719.15	− 66.54
CD40	ocu-miR-326-5p	MSTRG.62967.1	− 69.13

The sequencing results are reported in Additional files 1 and 2.

### Immunofluorescence analysis

Frozen vascular tissues from the different groups were allowed to stand for 1 h. The sections were rinsed in PBS three times. The cells were fixed with 4% paraformaldehyde for 25 min at room temperature and washed three times in 0.1 M DEPC-treated PBS. The tissues were then cut into 8-µm sections, which were routinely dehydrated, dipped in wax, embedded in paraffin, and sectioned. The paraffin tissue sections were routinely dewaxed and dehydrated with gradient alcohol. The sections were treated with diluted pepsin in 3% fresh citrate buffer at 37 °C for 1 min and then washed. The sections were removed by washing 3 times in sterile 0.5 M PBS and once in sterile distilled water. The hybridization cassette was placed with 20% glycerol at the bottom to maintain humidity. We added 20 µl of preliminary hybrid liquid to each section and incubated them for 3 h at 37 °C. The excess liquid was removed without washing. Each section was hybridized with 20 µl of hybridization probe at 37 °C overnight in incubators. Two (2) SSC washes, one 0.5 SSC wash and one 0.2 SSC wash was applied to each section for five, fifteen, and 15 min at 37 °C. Next, 40 µl of blocking solution was dropped onto the section, which was then sealed for 30 min at room temperature. Excess liquid was removed without washing. Afterwards, the sections were washed in PBS for 5 min and then incubated at 37 °C for 60 min with biotinylated mouse anti-digoxin (1:500; Boster). Slides were treated with SABC-FITC for 20 min and washed with 0.5 mol/l PBS 3 times for 5 min. The sections were stained with DAPI. The sections were then mounted with Slow Fade Light Antifade reagent (Molecular Probes, Invitrogen, CA, USA) and visualized with an Axioscope 2 Plus fluorescence microscope (Carl Zeiss, Inc., Germany). Assay kits were provided by Wuhan Boster Bioengineering Co., Ltd. The lncRNAMSTRG.22719.16 probe was designed by Shenzhen Jima Gene Co., Ltd. (Shenzhen, China).

### Dual-luciferase reporter assay

A total of 5*1000 293 T cells were seeded in each well of 96-well plates. The wild type (WT) sequence and mutant type (MUT) sequence of pmirGLO-CD40 mRNA (Jima Gene Co., Ltd., Shenzhen, China) were used. The WT sequence and MUT sequence of pmirGLO-lncRNA MSTRG.22719.16 RNA fragment were artificially synthesized (Jima Gene Co., Ltd., Shenzhen, China). Negative control (NC), ocu-miR-326-5p mimics, inhibitor negative control (INC), ocu-miR-326-5p mimic inhibitor, pmirGLO-CD40 (Wt)/(Mut), and pmirGLO-lncRNAMSTRG.22719.16 (Wt)/(Mut) were provided by Promega (Shenzhen, China) and transfected into cells with Lipo2000 (Invitrogen, Jinan, China) in accordance with the protocol. For transfection, Lipofectamine 2000 (Invitrogen) and 0.2 µg plasmid were used. They were cotransfected into 293 T cells following experimental grouping. The pmirGLO vector was used as a control. Transfected cells were maintained at 37 °C with 5% CO2 for 24 h. Cells were lysed 24 h after transfection. The Dual Luciferase Reporter Assay kit (Yeasen, Shanghai, China) was used to detect luciferase activity according to the instructions.

### Data analysis

The experiments were conducted three times each. The results are expressed as the mean ± standard deviation. A t test was used for comparisons between groups. Statistical analysis was performed using SPSS software (version 17.0). *P *≤ 0.05 was considered statistically significant. Significance is indicated as follows: *, *P *≤ 0.05; **, *P *≤ 0.01; and ***, *P *≤ 0.001.

## Results

### Characterization of PMMA and ES-PMMA by electron microscopy

Animals in the M group were treated with 8000 AXa IU enoxaparin sodium mixed with 40 g PMMA powder. The sample was observed under an electron microscope, and the gaps between the particles on the surface of ES-PMMA were filled with a syrup-like substance, which was considered the attached enoxaparin.

### Analysis of thrombus in different samples

Four hours after the establishment of the animal model, the thrombotic weight was 0.00706 ± 0.00136 g/cm in the Con group and 0.00551 ± 0.00115 g/cm in the M group [5]. Thrombogenesis showed a decreasing trend in the M group versus the Con group.

### Differential expression of thrombus-associated proteins in endothelial cells

**Fig. 3:**
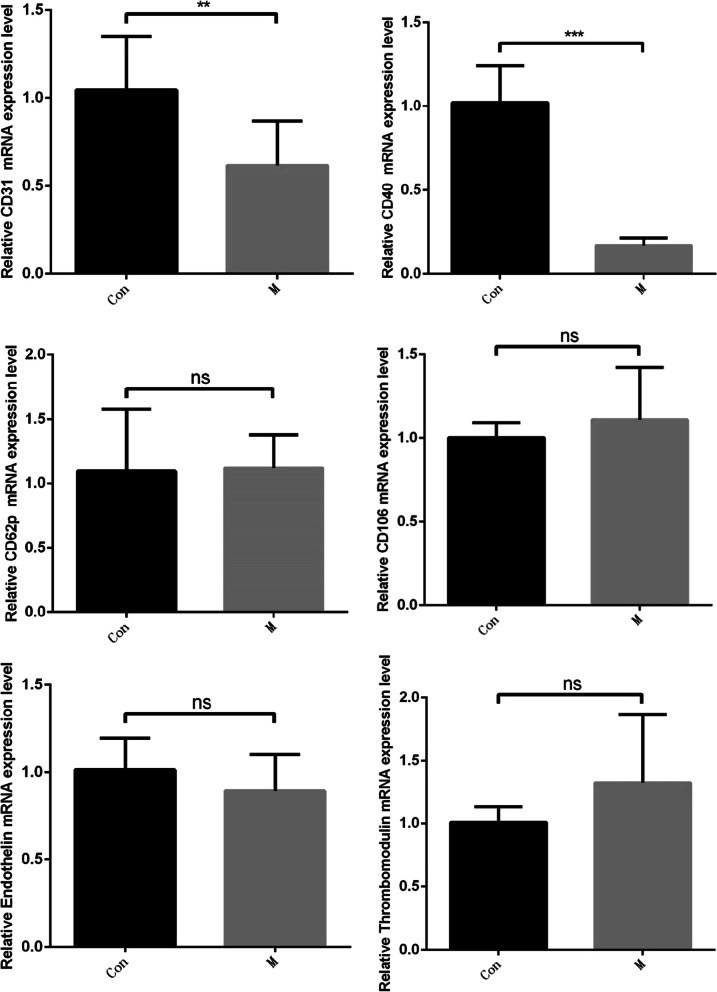
The mRNA expression levels of CD31, CD40, CD62p, CD106, endothelin, and thrombomodulin showed that CD31 and CD40 expression was decreased in the M group compared with the Con group (*P *< 0.01) and that CD40 mRNA expression was significantly decreased in the M group compared with the Con group (*P *< 0.001)

Vascular endothelial cells were collected for RT-qPCR and Western blotting. Relevant studies have shown that thrombosis is related to inflammation, a hypercoagulable state, vascular endothelial cell functional regulation or injury. Proteins related to thrombosis, mainly CD62p, CD31, CD106, CD40, endothelin, and thrombomodulin, are expressed in vascular endothelial tissue. We measured the mRNA expression levels by RT-qPCR. The results are shown in Fig. 3. We found that the expression of CD31 and CD40 was significantly decreased in the M group (*P *< 0.01) and that the expression of CD40 was significantly decreased in the M group (*P *< 0.001). There was no significant difference in other indexes (*P *> 0.05).

**Fig. 4:**
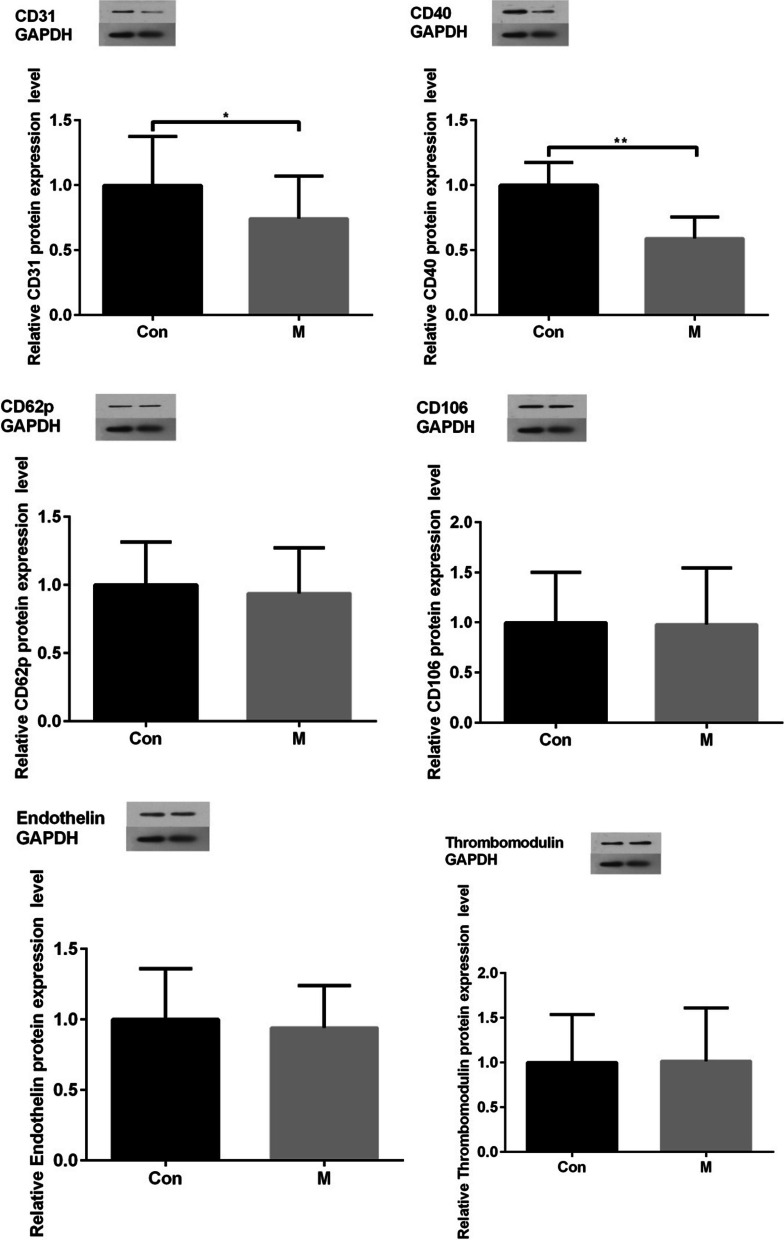
Protein expression levels of CD31, CD40, CD62p, CD106, endothelin, and thrombomodulin. The expression levels of CD31 and CD40 were decreased in the M group compared with the Con group (*P *< 0.05), and the expression levels of CD40 were significantly decreased in the M group compared with the Con group (*P *< 0.01)

The expression of these proteins was assessed by Western blotting. As shown in Fig. 4, the protein expression of CD40 was significantly decreased in the M group compared with the Con group (*P *< 0.01), and the protein expression of CD31 was decreased in the M group compared with the Con group (*P *< 0.05). There was no significant difference in other indexes between the two groups.

### The lncRNA expression in thrombotic samples was analysed through high-throughput sequencing and the ceRNA hypothesis of the CD40 gene.

**Fig. 5:**
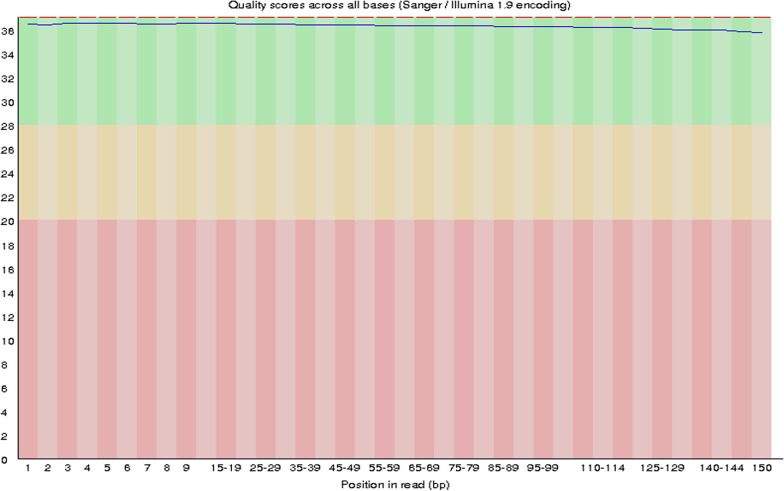
The average *Q* values of single individual box plots

**Fig.6:**
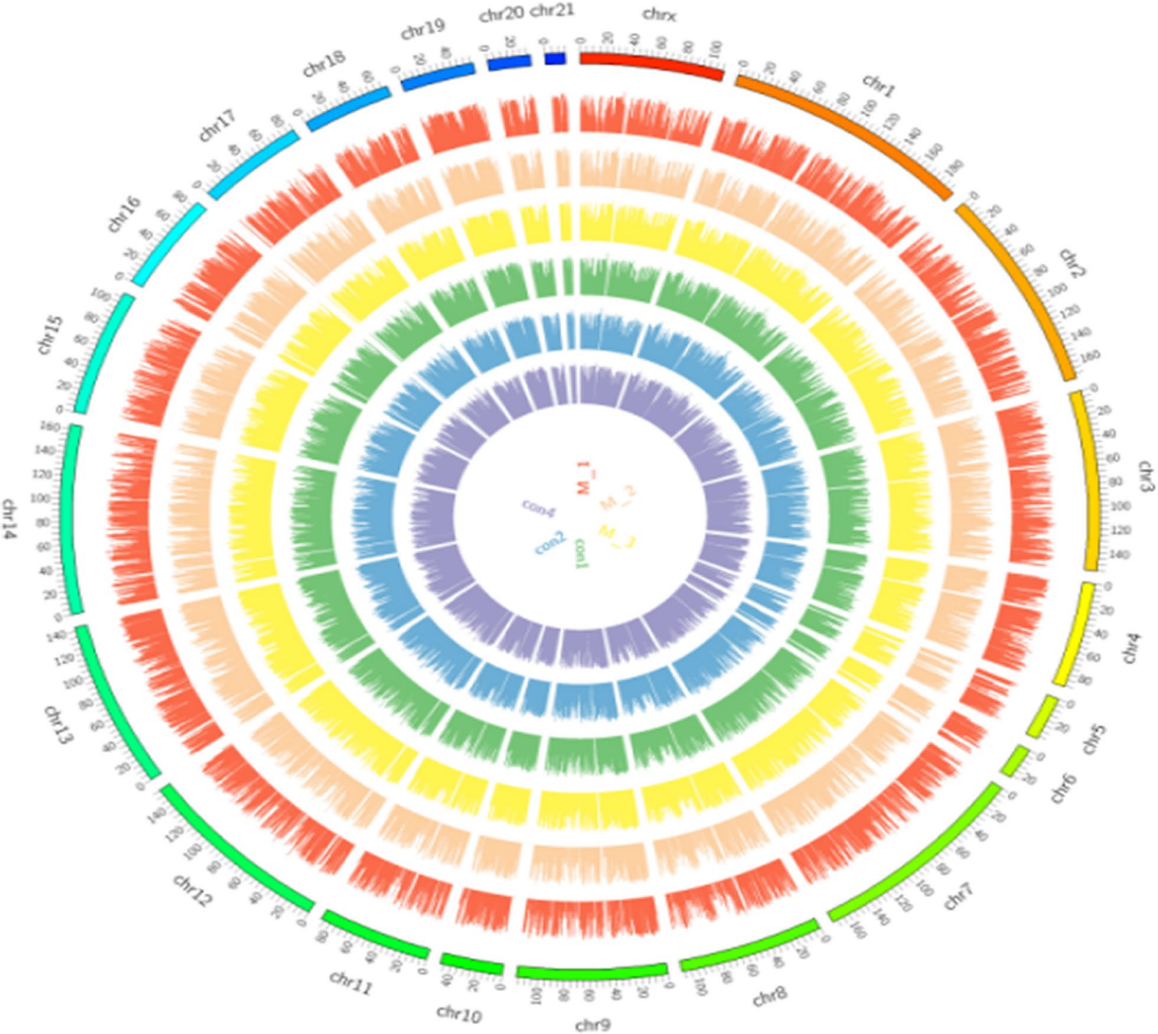
Distribution of sample sequence coverage

**Fig. 7:**
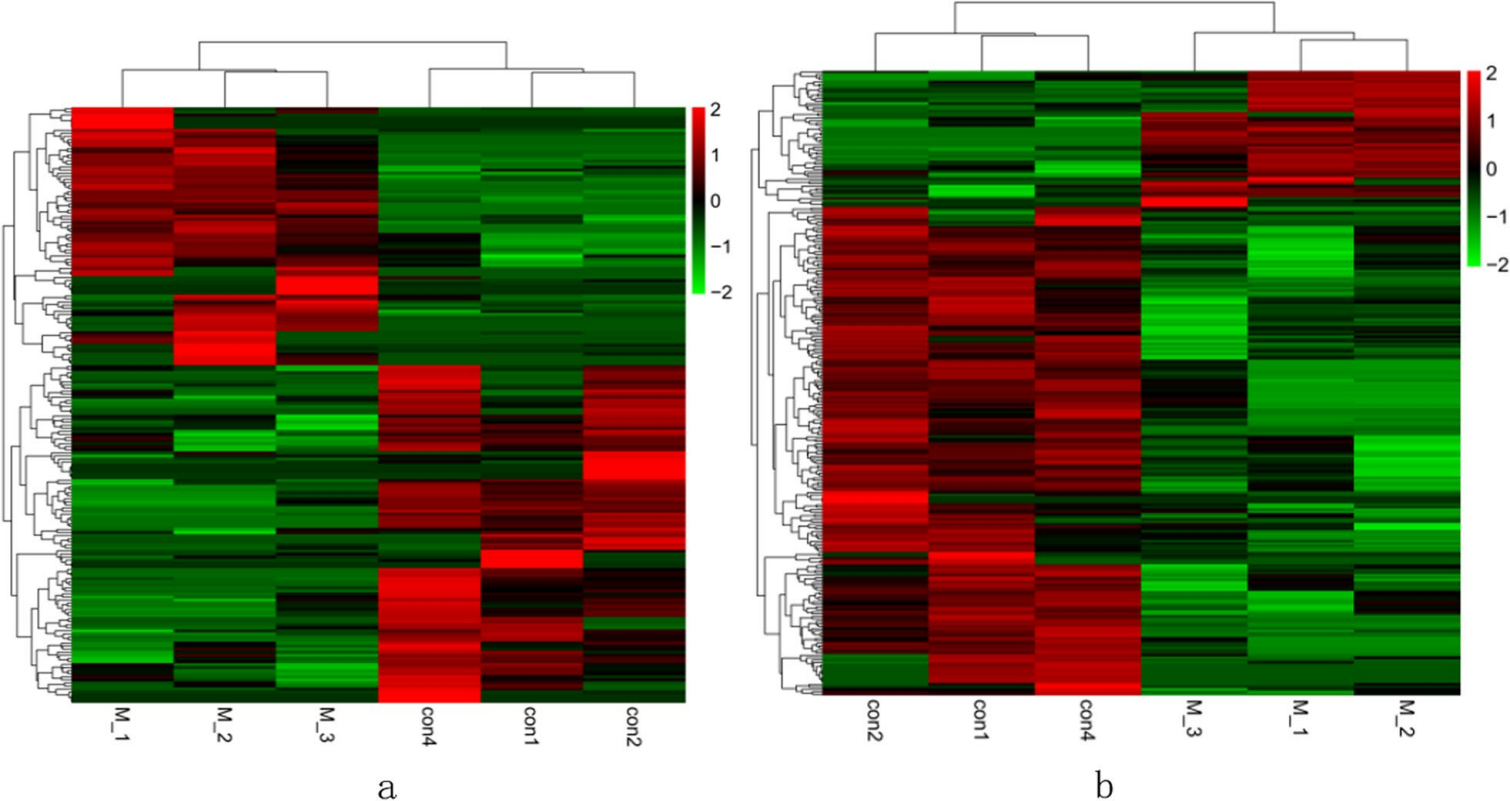
**a**: Heat map of differentially expressed total lncRNAs, **b** Heat map of newly discovered lncRNAs

**Fig. 8:**
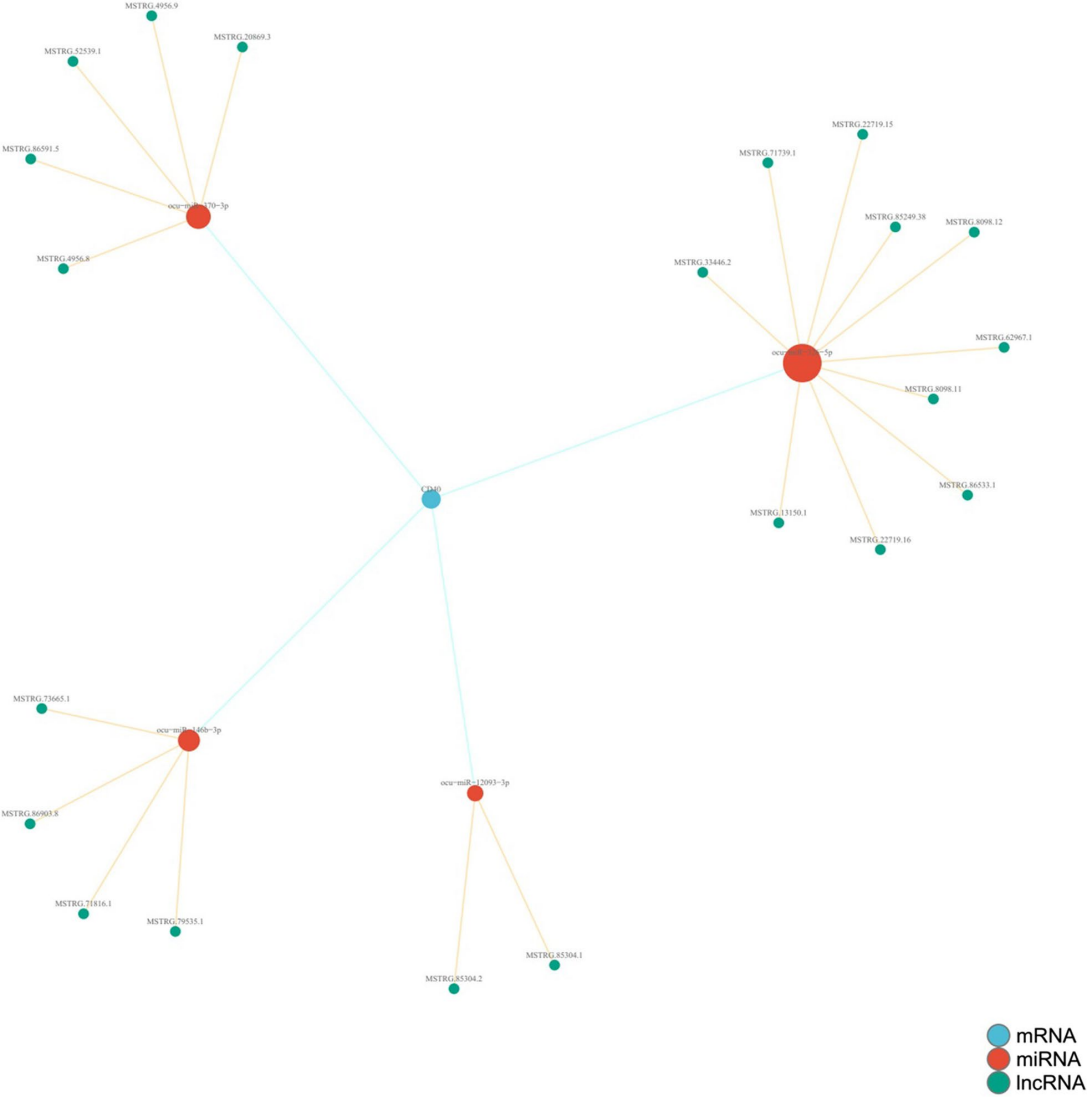
CD40 gene with the ceRNA hypothesis

As described above, high-throughput lncRNA gene sequencing analysis was carried out with pooled samples. The quality assessments are shown in Fig. 5. Figure 6 shows the distribution of the different genomes of coverage. Figure 7 shows that the total lncRNA expression profiles were characterized, and the expression of new lncRNAs that were differentially expressed was analysed. Differential expression of new genes was identified and is shown in Additional files 1 and 2. Among these differentially expressed lncRNAs, 85 lncRNAs were overexpressed, and 111 lncRNAs were downregulated. The ceRNA regulatory network was established and identified the CD40 gene based on the ceRNA hypothesis, as shown in Fig. 8 and Table 1.

**Fig. 9:**
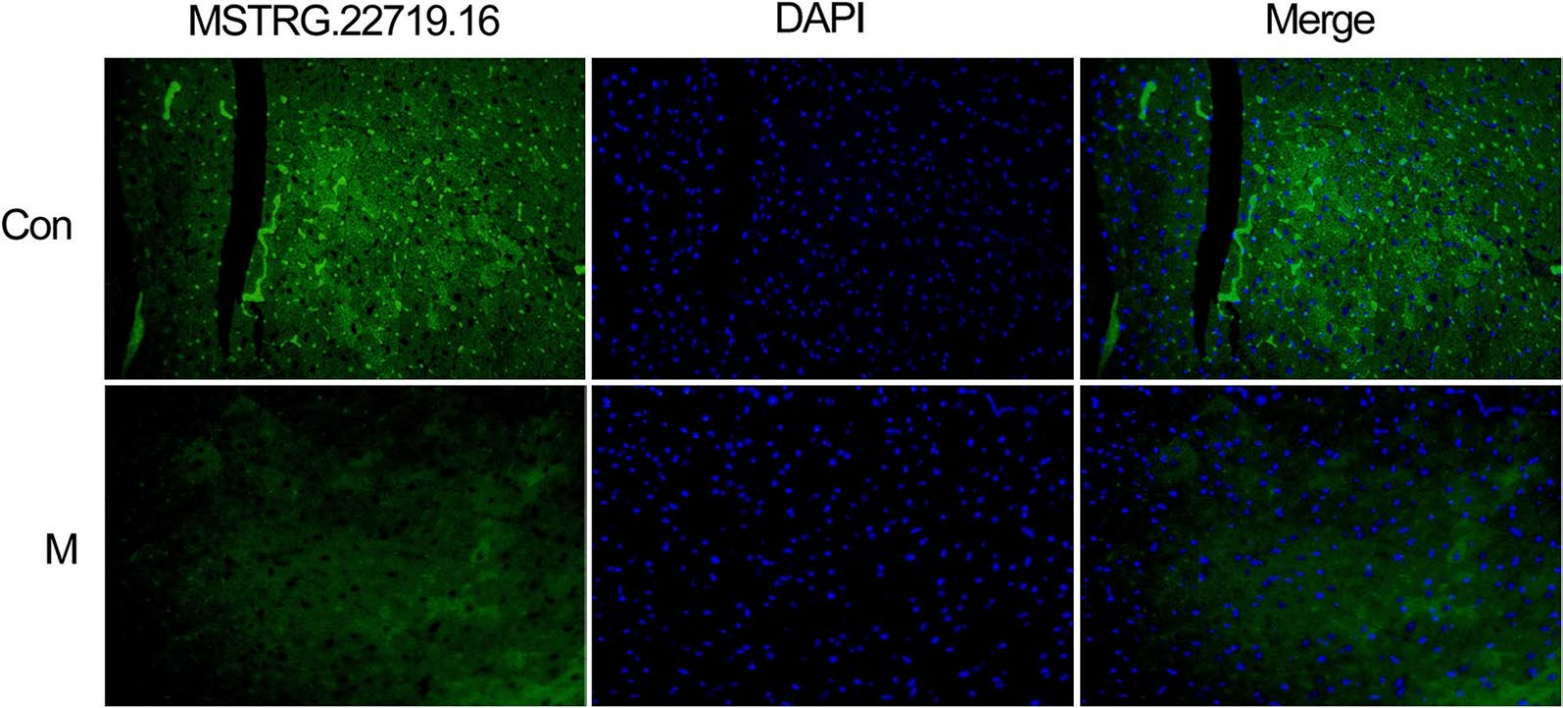
Low lncRNA MSTRG.22719.16 fluorescent probe expression in the M group in the figure

FISH experiments are shown in Fig. 9, base magnification: 200 × .

### Dual-luciferase reporter assay results

**Fig. 10:**
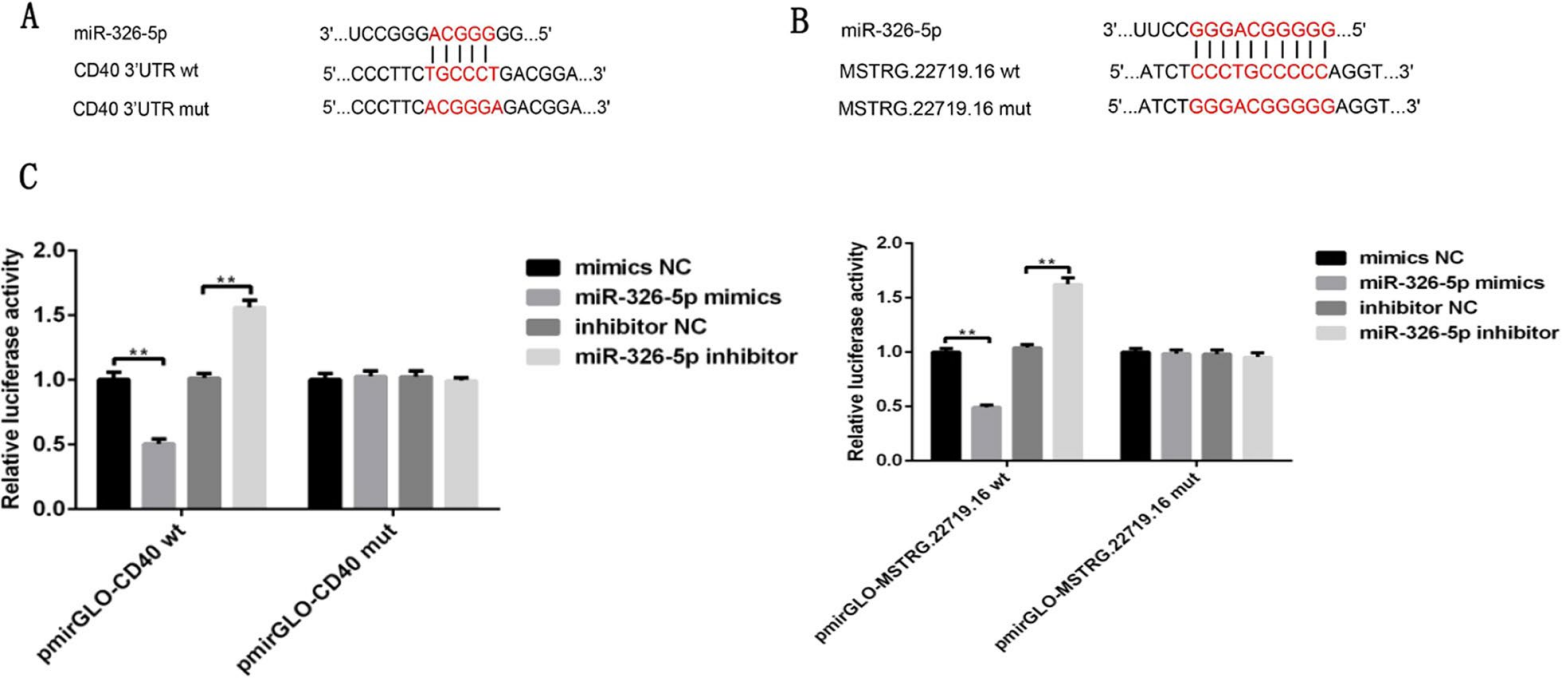
**A** Schematic of the ocu-miR-326-5p binding site in the 3′UTR of CD40 mRNA and the corresponding mutation. **B** Schematic of the ocu-miR-326-5p binding site in the partial base sequence of lncRNA MSTRG.22719.16 and the corresponding mutation. **C** pmirGLO-CD40 (Wt)/(Mut), pmirGLO-lncRNAMSTRG.22719.16(Wt)/(Mut) and negative control (NC), ocu-miR-326-5p mimics, inhibitor negative control (INC), and ocu-miR-326-5p mimic inhibitor were cotransfected into 293 T cells, and luciferase activity was determined, *P < 0.05, **P < 0.01. The results showed that the 3′UTR of CD40 mRNA and the base sequence of lncRNA MSTRG.22719.16 were able to bind to ocu-miR-326-5p mimics

Dual-luciferase reporter assay results see Fig. 10.

## Discussion

The application of bone cement in clinical practice is accompanied by BCIS [2, 30] which has an incidence rate that can reach 28%. The main causes of BCIS are embolisms caused by bone cement implantation, venous embolism in the lower limbs, and pulmonary emboli [31].

The local surgical environment was simulated in this study. Since bone cement is dispersed in the bone-prosthesis interface after hip and knee replacement and it is very difficult to collect samples around the bone cement, we simulated the surgical microenvironment during bone cement implantation. During joint replacement, the small blood vessels and arteriovenous around the joint are removed, and the small blood vessels in the medullary cavity and the blood circulating contact bone cement are removed. The main reasons for thrombosis include a slow endovascular blood stream, high blood condensation, platelet activation, abnormal expression of thrombosis-related regulatory proteins and endothelial cell injury [32–36]. Due to its physicochemical characteristics, bone cement can affect the blood vessels around the cement. We established an extracorporeal carotid artery shunt and then implanted a silk thread covered with bone cement to simulate the microenvironment in which blood flows through the surface of bone cement. In this way, bone cement can contact the inner wall of blood vessels, and then, we can assess the influence of bone cement on local blood vessels. Using this model, we were able to quantitatively evaluate thrombosis. Further research on the exact molecular mechanisms of thrombotic formation is necessary. Because the thrombus attached to bone cement was the direct product after the action of various factors, as well as the complex substance regulated by many factors, different lncRNA prediction software programs were used for sequencing the thrombosis samples, and detailed studies are necessary to clarify the molecular mechanisms.

Thrombosis is mainly caused by platelet activation, blood hypercoagulability, injury of vascular endothelial cells and abnormal expression of thrombosis-related proteins [28, 32–35]. Bone cement causes local thrombosis by acting on vascular endothelial tissue or directly contacting blood components. We centralized this model in which bone cement stimulates all kinds of arteriovenous microvascular and intramedullary blood vessels around the joint replacement. We used a larger vessel to stimulate various small blood vessels, such as capillary arteries and veins, knee and periarticular arteries and veins. One of these points reflects the overall environment of the operation.

In this study, vascular tissues in contact with bone cement were selected to compare the influence of PMMA and ES-PMMA on the expression of thrombosis-related proteins by measuring the expression of these proteins. Among the proteins expressed by endothelial cells, endothelin [11], CD106 [13], CD62p [14, 15], CD31 [16], thermoregulatory protein [19, 20] and CD40 [21, 22] are closely related to thrombosis. We assessed the differential expression of thrombus-related proteins and found that the expression of CD40 in the ES-PMMA group was significantly lower than that in the PMMA group. In addition, previous studies have proven that CD40 has a regulatory effect on thrombosis [37]. The activity of enoxaparin sodium is ascribed to anticoagulant factor Xa [38] which is a serine protease [39]. Serine proteases exist in the complement system and are digestive enzymes [40, 41]. The complement system is composed of inherent complement components, complement receptors and complement regulatory proteins [42]. Some complement components are expressed in vascular endothelial cells [43] and CD40 is also expressed in vascular endothelial cells [44].

The ceRNA hypothesis has led to a novel way of studying lncRNA‒miRNA-mRNA crosstalk and has received widespread attention. Many bone and joint researchers have studied this mechanism [45–48]. The same analysis can be performed to identify the molecular mechanisms of regulating CD40 expression. To clarify the effect of different materials on thrombosis and lncRNA expression profiles, we generated lncRNA expression profiles through a high-throughput sequencing platform. This work identified novel differentially expressed sequence-specific lncRNAs in the ES-PMMA group. A ceRNA regulatory network directly associated with CD40 was generated based on gene sequence analysis and alignment. The regulatory pathway of lncRNA MSTRG.22719.16/ocu-miR-326-5p/CD40 was predicted. Scholars have conducted extensive research on miR-326-5p. This molecule plays a regulatory role in hepatocyte metabolism, bone metabolism, apoptosis and myocardial infarction [49–52]. After the sequence was aligned in this research, we predicted regulatory binding sites for CD40 mRNA in its base sequences and found that it plays a role in competitive inhibitory regulation. LncRNA MSTRG.22719.16 was found to have a binding site with ocu-miR-326-5p by gene sequence alignment. Additionally, the direct binding of ocu-miR-326-5p to the 3′-UTR of CD40 mRNA and lncRNA MSTRG.22719.16RNA was validated using a dual luciferase reporter assay. As shown by FISH, lncRNA MSTRG.22719.16 was expressed at lower levels in vascular tissues of the M group and exercised its biological regulatory function in vascular tissues. CD40 is only expressed in endothelial cells in vascular tissues. Furthermore, the mechanism of the lncRNA MSTRG.22719.16/ocu-miR-326-5p/CD40 regulatory pathway was elucidated.

## Conclusion

In summary, we found that the novel material ES-PMMA can reduce local thrombosis through the lncRNA MSTRG.22719.16/ocu-miR-326-5p/CD40 axis by using a simple and reproducible animal model, high-throughput sequencing techniques and the ceRNA mechanism.

### Supplementary Information

**Additional file 1.** The meaningful up-regulated results of sequencing.

**Additional file 2.** The sequencing results of sample.

## Data Availability

The datasets used and/or analysed during the current study are available from the corresponding author on reasonable request.

## Abbreviations

PMMA: Polymethylmethacrylate
ES: Enoxaparin sodium
SEM: Scanning electron microscopy
FISH: Fluorescent in situ RNA hybridization
